# Loss of luminal carbonic anhydrase XIV results in decreased biliary bicarbonate output, liver fibrosis, and cholangiocyte proliferation in mice

**DOI:** 10.1007/s00424-021-02659-3

**Published:** 2022-02-04

**Authors:** Zhenzhen Zhou, Jiajie Qian, Archana Kini, Brigitte Riederer, Dorothee Römermann, Gerolf Gros, Ursula Seidler

**Affiliations:** 1 Department of Gastroenterology, Hannover Medical School, Hannover, Germany; 2 Present Address: Department of Gastroenterology, Tongji Hospital, Tongji Medical College, Huazhong University of Science and Technology, Wuhan, China; 3 Present Address: Department of Gastrointestinal Surgery, First Affiliated Hospital, College of Medicine, Zhejiang University, Hangzhou, China; 4 Department of Molecular and Cell Physiology, Hannover Medical School, Hannover, Germany; 5 Department of Gastroenterology, Hepatology and Endocrinology, Hannover Medical School, Carl Neuberg Straße 1, 30625 Hannover, Germany

**Keywords:** Biliary bicarbonate umbrella, Bile acids, Bicarbonate, Liver, Acid–base balance, Carbonic anhydrases, Electrolyte transport, Sclerosing cholangitis

## Abstract

Carbonic anhydrase XIV (Car14) is highly expressed in the hepatocyte, with predominance in the canalicular membrane and its active site in the extracellular milieu. The aim of this study is to determine the physiological relevance of Car14 for biliary fluid and acid/base output, as well as its role in the maintenance of hepatocellular and cholangiocyte integrity. The common bile duct of anesthetized *car14*^−/−^ and *car14*^+*/*+^ mice was cannulated and hepatic HCO_3_^−^ output was measured by microtitration and bile flow gravimetrically before and during stimulation with intravenously applied tauroursodeoxycholic acid (TUDCA). Morphological alterations and hepatic damage were assessed histologically and immunohistochemically in liver tissue from 3- to 52-week-old *car14*^−/−^ and *car14*^+*/*+^ mice, and gene and/or protein expression was measured for pro-inflammatory cytokines, fibrosis, and cholangiocyte markers. Biliary basal and more so TUDCA-stimulated HCO_3_^−^ output were significantly reduced in *car14*^−/−^ mice of all age groups, whereas bile flow and hepatic and ductular morphology were normal at young age. *Car14*^−/−^ mice developed fibrotic and proliferative changes in the small bile ducts at advanced age, which was accompanied by a reduction in bile flow, and an upregulation of hepatic cytokeratin 19 mRNA and protein expression. Membrane-bound Car14 is essential for biliary HCO_3_^−^ output, and its loss results in gradual development of small bile duct disease and hepatic fibrosis. Bile flow is not compromised in young adulthood, suggesting that Car14-deficient mice may be a model to study the protective role of biliary canalicular HCO_3_^−^ against luminal noxi to the cholangiocyte.

**Supplementary Information:**

The online version contains supplementary material available at 10.1007/s00424-021-02659-3.

## Introduction

Carbonic anhydrases (CAs) are a family of functionally related proteins that catalyze the reaction H^+^  + HCO_3_^−^  ↔ CO_2_ + H_2_O, albeit with highly variable enzymatic activity. The mammalian organism expresses 11 enzymatically active α-CAs, some of which are ubiquitous but with strongly variable expression levels, while others are highly organ- or cell-type-specific [29, 37, 39]. The localization is either cytoplasmic, organellar, or membrane-bound, and different types of membrane association have been described. Both cytoplasmic and membrane-bound CAs are important for the supply of protons or base to acid/base transporters, and may increase their transport rates several fold if existing in a “HCO_3_^−^ transport metabolon” with the respective transporter [34]. In addition, membrane-bound carbonic anhydrases may help to dissipate proton gradients at the intra- as well as extracellular membrane and thus improve buffering both in the intra- and extracellular space [7]. Lastly, a mere increase in the pool of available protons/base moieties may also increase the transport rate of acid/base transporters[10, 36].

Carbonic anhydrase XIV (CAXIV, Car14) is one of the most recently identified CA with predominant expression in the brain, kidney, liver, skeletal muscle, heart, and lung [19]. Full-length Car14 is a transmembrane protein composed of an extracellular catalytic domain, a single transmembrane helix, and a short intracellular polypeptide segment [1, 52]. Car14 is expressed in the hepatocyte with canalicular membrane predominance, but also with basolateral localization, supposedly with its active site in the extracellular milieu [38, 52]. Its role for hepatocyte function has not been studied. However, a 4-week application of the dual farnesoid receptor agonist INT-767 was tested in the *mdr2*^−/−^ mouse model, in which the absence of the canalicular phospholipid flippase MDR2 (MDR3 in humans) results in a decrease in mixed micelles and an increase of monomeric bile acid, followed by bile acid–mediated hepatic injury [18, 19, 52]. The treatment was associated with both a decrease in bile acid synthesis, an increase in HCO_3_^−^ rich choleresis, an increase in Car14 expression, and significant hepatoprotection [6]. These interesting data suggest that the function of Car14 in hepatobiliary acid/base balance and hepatoprotection warrants further study.

Early studies of hepatic acid/base transport and bile secretion have employed isolated liver perfusion [30, 42], isolation of canalicular membrane vesicles [35], and the isolation of hepatocyte couplets (in which a distinct basolateral and canalicular membrane is preserved) to investigate the expression and importance of acid/base transporters in hepatocyte homeostasis and their involvement in bile formation [22, 48]. Since then, a multitude of transport proteins for a large variety of organic anions and cations have been identified in the canalicular membrane, some of them coupled to the transport of protons, in addition to Na^+^/H^+^ and Cl^−^/HCO_3_^−^ exchange proteins [11, 12] suggesting that a canalicular membrane–bound carbonic anhydrase may enhance proton gradient dissipation, CO_2_ recycling, and base flux by functionally interacting not only with Cl^−^/HCO_3_^−^ exchangers but also with an organic anion transporters [7, 18]. Basolateral acid extruders like Na^+^/H^+^ exchangers and base uptake mechanisms like Na^+^/HCO_3_^−^ cotransporters have also been described in hepatocytes [32] and functionally linked to bile acid–dependent bile flow and hepatic HCO_3_^−^ output [3, 30, 42]. Car14 expression is also evident in the hepatocyte sinusoidal membrane [38], and may play a role in providing HCO_3_^−^ and removing extruded protons from the extracellular leaflet, thus aid in the maintenance of an alkaline intracellular pH_i_, a requirement of biliary HCO_3_^−^ output.

We therefore investigated basal and tauroursodeoxycholic acid (TUDCA)–stimulated biliary fluid and HCO_3_^−^ secretion in anesthetized *car14*^−*/*−^ mice and *car14*^+*/*+^ littermates at different age. We also studied liver histology and measured protein and/or gene expression for pro-inflammatory cytokines and pro-fibrotic and proliferative markers in the livers of mice from 3 to 52 weeks/age.

## Materials and methods

### Animals

All mice were bred on the C57BL/6 background in the animal facility of Hannover Medical School (MHH). Mice were maintained with controlled light/dark cycles and free access to water and food. All experiments were approved by the Local Institutional Animal Care and Research Advisory Committee at the Hannover Medical School and authorized by the Niedersächsisches Landesamt für Verbraucherschutz und Lebensmittelsicherheit (LAVES) (TVA Nr. 33–12-42,502–04-15–1847). The experimental procedures performed and the type of anesthesia used were according to university and national guidelines and are explained below. The *car14*^−/−^ (B6.129S1-Car14^tm1sly^) mice were originally created in the group of William Sly in the Edward A. Doisy Department of Biochemistry and Molecular Biology [45] and provided by Prof. Gerolf Gros, Institute of Physiology, MHH. For the experimental groups, similar number of female and male mice were used.

### Reagents

TUDCA (Tauroursodeoxycholic acid) (Calbiochem/MerckBiosciences), Mayer’s hematoxylin, Eosin Y solution, Picric acid, Direct Red 80, 30% H_2_O_2_, EDTA (Sigma-Aldrich), Xylene (J.T. Baker GmbH), Permount, Fast Green FCF (Thermo Fisher GmbH), Goat Serum (Vector Laboratories), anti-CK19 antibody (DSBH of Iowa University), AEC (red) substrate Kit, phosphate-buffered saline, goat anti-mouse IgG (Life Technologies GmbH). All other chemicals were obtained from Applichem GmbH, Germany, unless mentioned otherwise.

### In vivo biliary secretion experiments

Mice were anesthetized by isoflurane (Forene; Abbott Germany, Wiesbaden, Germany) under spontaneously breathing. After induction of anesthesia, the mice received tracheal intubation, and mechanical ventilation was initiated with an anesthesia unit which was constituted of an isoflurane pump (Univentor 1250 Anaesthesia Unit; AgnTho, Lidingö, Sweden) and a ventilator (MiniVent Type 845; Hugo Sachs Electronik, March-Hugstetten, Germany). After anesthesia, the mice received 50 mg/kg metamizol in 50 µl H_2_O. The isoflurane pump supplied mixed narcotic gas (mixture of ∼10–15% oxygen, ∼85–90% air, and 2.0 ± 0.2% isoflurane) to the ventilator. Mice were ventilated mechanically at a rate of 120–160/min with a tidal volume of 6–8 ml/kg body weight and kept on a rectal thermistor-controlled heating pad, to maintain the core body temperature between 37 and 38 °C, for the duration of the surgery and the experiment. A catheter was then placed into the left carotid artery and was connected with a blood pressure transducer operating with PowerLab system (AD Instruments, Hastings, UK), for a continuous monitoring of blood pressure and continuous injection. An alkaline solution was infused into carotid artery at a rate of 0.1 ml/h to correct the systemic acid–base balance as following composition: 200 mM Na^+^, 100 mM CO_3_^2−^, 5 mM K^+^, and 5 mM Cl^−^. The left jugular vein was also intubated for infusion of TUDCA dissolved in PBS at the rate of 0.2 ml/h PBS and 600 nmol/min TUDCA [14, 40]. The mouse was placed under the operating microscope (Wild M3Z, Wetzlar, Germany), and the abdomen was opened with a short (2–3 cm) mid-ventral celiotomy and the neck of the gallbladder was ligated with a suture. The common bile duct was then cannulated with a polyethylene tubing made very thin at the tip over a flame, for continuous collection of bile. Finally, the abdominal cavity was closed with a continuous suturing and the animal was allowed to rest for $\sim$ 20 min, before the start of the experiment. Baseline values were collected for 40 min, after which the mice were infused with TUDCA via carotid vein for another 60 min. Mice were killed by cervical dislocation at the end of the collection period. Livers were excised, weighed, dissected, and processed for histology and laboratory examinations.

During the experiment, blood pressure was continuously monitored and if too low, either the isoflurane concentration was adjusted, or the infusion speed of a Ringer electrolyte solution via the vein was increased, or both. Sequential blood samples were taken for blood gas analysis and adjustments were made by increasing the infusion speed, or by giving a solution with less NaCO_2_ (rarely necessary). The sample for blood gas was taken from the carotid artery into a heparinized glass capillary (Clinitubes, Radiometer, Copenhagen, Denmark) during the experiment if necessary. The blood sample was analyzed in a Radiometer blood gas analyzer (ABL-5 Blood Gas Analyzer, Radiometer, Copenhagen, Denmark) immediately before coagulation.

### Measurement of biliary bicarbonate secretion

The rate of luminal alkalinization was determined via back titration of the bile sample to pH 4.5 with 5 mM HCl under continuous N_2_ gassing using pH–stat equipment (PHM82 Standard pH meter, Radiometer, Copenhagen, Denmark) [46, 47]. The pH electrode was routinely calibrated with standard buffers before the initiation of the titration. The amount of titrated HCl was considered equivalent to the biliary bicarbonate secretion. The rates of bile alkalinization are expressed as micromoles of the base secreted per hour (µmol/h).

### Measurement of bile flow

Bile flow was measured gravimetrically. Bile juice was collected in a pre-weighed plastic tube and was weighed again, immediately after the end of the collection period. The difference in measured weight was considered the bile flow for that collection period. All volume measurements were calculated from weight, with the density of normal saline set arbitrarily to 1.0. The rates of bile flow are expressed as microliter of the base secreted per hour (µl/h).

We also evaluated the effect of i.v. secretin (17 nmol/kg porcine secretin as bolus injection into the vein) stimulation on bile flow and biliary HCO_3_^−^ secretion, which stimulates via exclusively on cholangiocytes expressed secretin receptors [26], both in young and in adult mice. While some stimulation of bile flow and HCO_3_^−^ output was observed after i.v secretin stimulation, the degree of stimulation over baseline was low (Jiajie Qian and Taolang Li, unpublished, 2014). This was in contrast to a strong stimulation of pancreatic ductal bicarbonate output, demonstrating that the secretin was biologically active and that the dose was sufficient [31]. Therefore, we did not consider it possible to study a selective effect of Car14 deletion on cholangiocyte-mediated HCO_3_^−^ output, and the major contribution of the TUDCA-stimulation HCO_3_^−^ output will originate from the hepatocytes.

### Liver tissue work-up

We followed the guidelines for standardized work-up for mouse models of the International PSC Study Group (IPSCSG) [17]. Liver tissue was collected immediately after the carotid dissection, at the conclusion of the experiment. It was flushed in 1X PBS solution, weighed, and was separated into different sections, for different analyses. The whole separating process was done in 1X PBS solution. For mRNA expression analysis, we used the whole lobe 3, and for Western analysis, we used the whole lobe 1, or 1 + 4, to avoid bias by purposely selecting the peripheral liver sections, which show stronger histological alterations, despite the fact that this may underestimate the inflammatory, fibrotic, and proliferative changes at the mRNA and protein expression level.

For routine histological stains, tissue was drop-fixed in 5% paraformaldehyde (PFA) for 24 h. The excess PFA was removed with PBS rinse; and the sample was put in 1X PBS at least 12 h after paraffin infiltration; the tissue was embedded into paraffin blocks which were cut into 2 µm sections.

### Histological and immunohistochemical analyses

H&E staining and Sirius Red staining were performed according to the suggestions of the manufacturer’s instructions (Sigma-Aldrich, USA). The CK19 immunohistochemical analysis was performed as described for Ki67 immunohistochemistry staining protocol, described by Vector Labs Company (Germany) with the following changes. Sections were placed in small metal containers, containing hot EDTA antigen retrieval buffer. The container was heated in a water bath for 30 min at 96–98 °C, blocked with 5% goat serum in PBST for 60 min, and incubated with anti-cytokeratin 19 (CK19) 1:200 overnight at 4 °C. The sections were then thawed at room temperature for 30 min, incubated with goat anti-rat IgG 1:500 for 60 min, and stained by AEC substrate Kit. Finally, the slides were mounted with medium.

### qPCR protocol

Total RNA was extracted from ≤ 50 mg mouse liver tissue using RNeasy Mini Kit (QIAGEN) according to the manufacturer’s instructions. For cDNA synthesis, 1 µg RNA was mixed with 50 ng Random Hexamer and 125 ng Oligo(dT)_18_ primers, 2 µl of 5 × M-MLV RT buffer, 100 units of M-MLV Reverse Transcriptase, 10 units of RiboLock RNase Inhibitor (all from Thermo Scientific), 2 µl of 10 mM dNTP-Mix (Bioline), and RNase-free H_2_O in 10 µl total volume. The mixture was then incubated at 25 °C for 10 min, 42 °C for 60 min for reverse transcription, and then 70 °C for 10 min to denature the enzyme in a thermal cycler (Applied Biosystems). cDNA was diluted 1:50 with DNase-free H2O for qPCR. Four microliters of diluted cDNA in combination with 5 µl 2X qPCRBIO SyGreen Mix Lo‐ROX (PCR Biosystems) and 0.5 µl of each forward and reverse primers at 10 µM concentration for the target genes (Supplementary Table. 1) was used for qPCR in a Rotor-Gene Q device (QIAGEN).

### Western analysis

Mouse liver tissue was homogenized in RIPA lysis buffer (25 mM Tris, 150 mM NaCl, 1% Nonidet P-40, 0.5% sodium deoxycholate, 0.1% SDS, pH 7.5) supplemented with a cocktail of protease inhibitors (40 µg/ml PMSF, 20 µg/ml leupeptin, 20 µg/ml pepstatin A, 20 µg/ml antipain, 4 mM benzamidin, 1 mM DTT) using an Ultra‑Turrax homogenizer (IKA) set for 3 times each 10 s by means of at speed 4, with 10 s intervals on ice. The homogenate was further cold-treated by 10 up and down strokes with a Potter homogenizer at 1000 rpm (Braun Biotech International) and the debris was sedimented by centrifugation at 10,000 rcf at 4 °C for 10 min. The protein content of the lysate was determined by the Bradford method (Bio-Rad). Thirty micrograms total protein was resolved by 10% SDS-PAGE (Mini PROTEAN Tera Cell system; Bio-Rad) and wet-transferred to PVDF membrane (Mini Trans-Blot Cell system; Bio-Rad) at 300 mA for 120 min. The membrane was then blocked in the TBS containing 0.1% Tween 20 and 5% non-fat milk (Bio-Rad). Primary antibodies against Cytokeratin 19 (clone TROMA-3; DSHB) at 1:5000 dilution or Vinculin (V9131, Sigma-Aldrich) at 1:10,000 dilutions were prepared in blocking buffer and exposed to the membrane overnight at 4 °C. After proper washing steps before and after incubating the membrane with HRP-goat anti-mouse secondary antibody (Thermo Scientific) at 1:10,000 dilution for 40 min at room temperature, the corresponding protein signals were visualized by ECL Western Blotting Detection Reagents (GE healthcare #RPN2209) and developed on an X-ray film. The bands were scanned and quantified using ImageJ software.

### Statistical analysis

Statistical analyses were performed using the Prism analysis program (GraphPad 8.0, San Diego, CA, USA). The statistical significance of data was tested via repeated-measures analysis of variance. To test differences within a group, one-way ANOVA was used followed by a Tukey post hoc test. Between groups, two-way ANOVA was used followed by a Bonferroni post hoc test. A *p*-value of less than 0.05 was considered significant. All data are presented as means ± standard error of the means (SEM).

## Results

### Biliary HCO_3_^−^ output in *car14*^*−/−*^ and *car14*^+*/*+^ littermates at different age

**Fig. 1:**
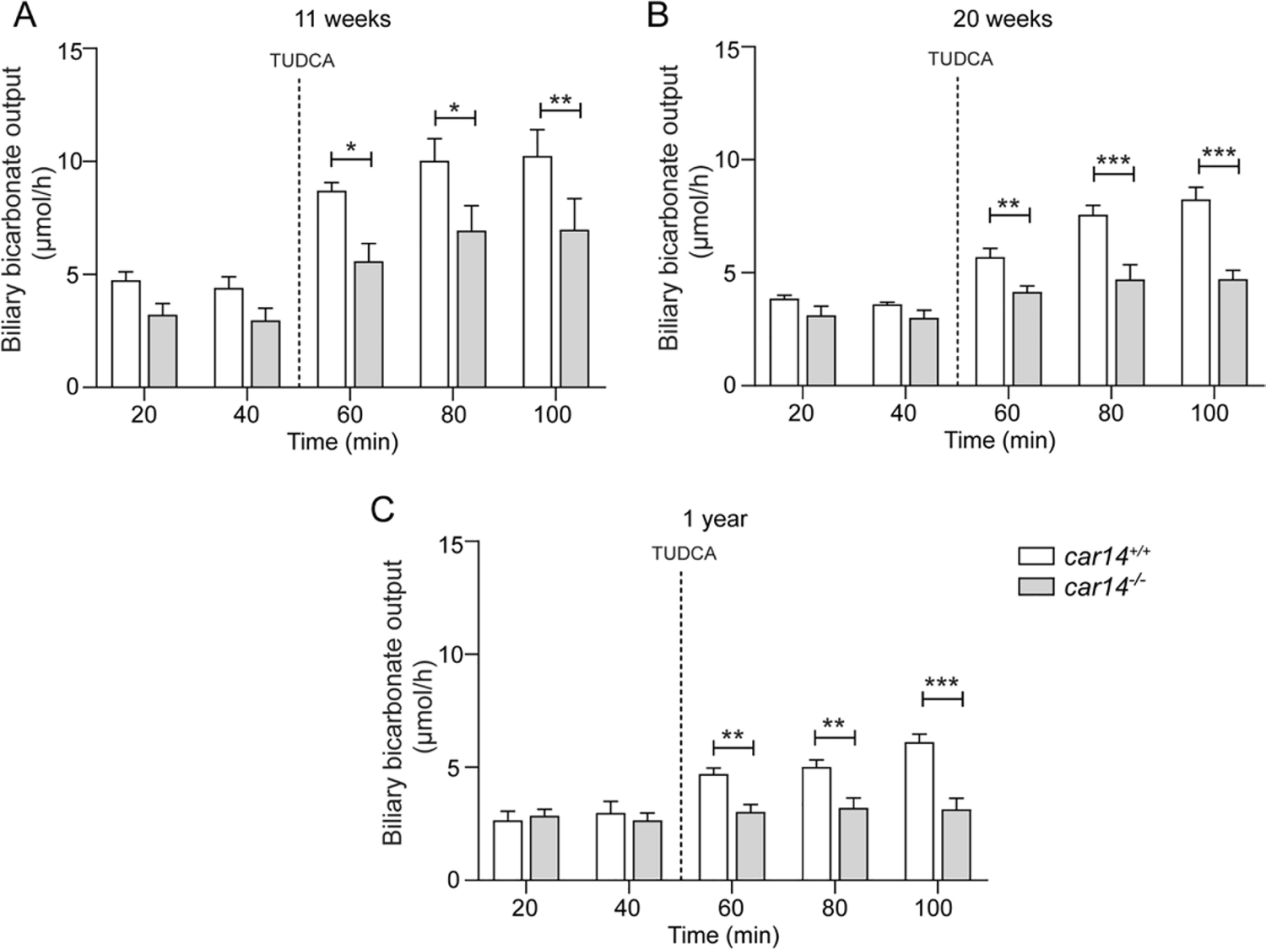
Biliary HCO_3_^−^ output in µMol/h in anesthetized *car14*^*−/*−^ mice aged 11 (**A**), 20 (**B**), and 52 (**C**) weeks, and the respective *car14*^+*/*+^ littermates. Both basal HCO_3_^*−*^ output and the degree of stimulation by TUDCA were higher in young compared to aged mice, in both genotypes. The TUDCA-stimulated biliary HCO_3_^*−*^ output was significantly reduced in *car14*^*−/−*^ compared to *car14*^+*/*+^ at all age groups (**A**–**C**). *n* = 4–8 in each group

Biliary HCO_3_^−^ output was measured in anesthetized *car14*^−/−^ mice aged 11, 20, and 52 weeks, and the respective *car14*^+*/*+^ littermates, by pH–stat titration of the sequentially collected bile samples over the course of the experiment. Biliary HCO_3_^−^ output was measured in µMol/h, and both basal HCO_3_^−^ output and the degree of stimulation by TUDCA were higher in young compared to aged mice, in both genotypes. The TUDCA-stimulated biliary HCO_3_^−^ output was significantly reduced in *car14*^−/−^ compared to *car14*^+*/*+^ at all age groups (Fig. 1A–C). TUDCA completely failed to stimulate biliary HCO_3_^−^ output significantly in 1-year-old *car14*^−/−^ mice (Fig. 1C).

### Bile flow was not different in young and adult *car14*^−*/*−^ and *car14*^+*/*+^ littermates and became compromised at advanced age

**Fig. 2:**
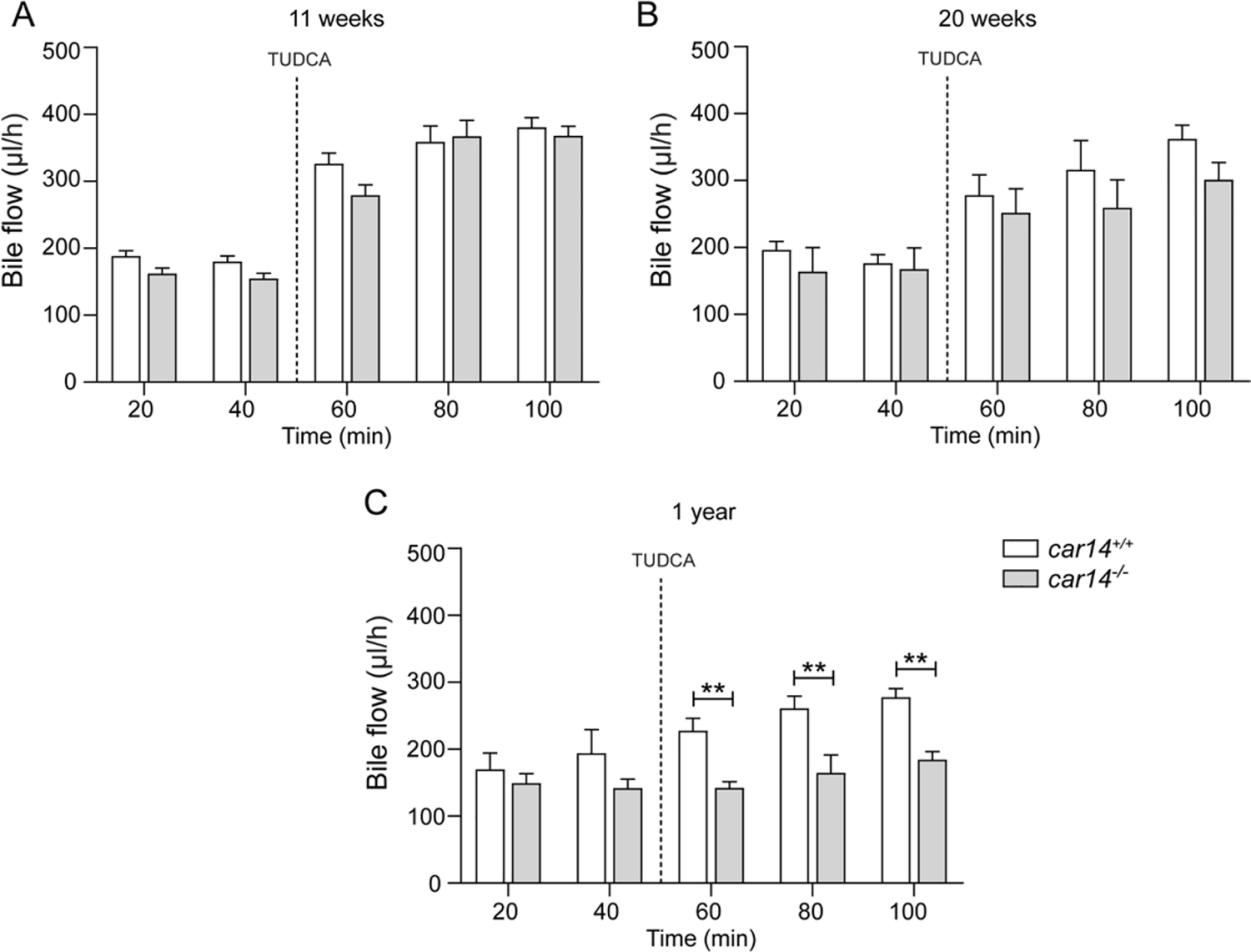
Biliary fluid output in the basal state and after TUDCA application (600 nmol/min intravenously) in *car14*^*−/−*^ mice and *car14*^+*/*+^ littermates, at 11 weeks (**A**), 20 weeks (**B**) and 52 weeks (**C**). In all mice, TUDCA application significantly stimulated bile flow (indicated by the # signs). In 11-week mice, no difference in bile flow rates was observed between *car14*^*−*/*−*^ and *car14*^+*/*+^ mice (**A**). In 20-week mice, TUDCA-induced bile flow was non significantly reduced in *car14*^*−*/*−*^ compared to *car14*^+*/*+^ mice, while it was significantly reduced in 1-year-old *car14*^*−*/*−*^ mice compared to the respective littermates (**B**, **C**). *n* = 4–8 in each group

Figure 2A displays a typical time course for biliary fluid output in the basal state and after TUDCA application (600 nmol/min intravenously) in *car14*^−/−^ mice and *car14*^+*/*+^ littermates at 11 weeks of age. While TUDCA application significantly stimulated bile flow, no difference was observed between *car14*^-*/*-^ and *car14*^+*/*+^ mice. These experiments demonstrate that the absence of Car14 interferes with a biliary alkalinization process that is not coupled to fluid secretion (i.e., a proton or base exchangers rather than anion channels, because HCO_3_^−^ secretion via the latter (either as a Cl^−^ conductance coupled to an anion exchanger, or via a Cl^−^/HCO_3_^−^ channel, or both) is accompanied by fluid secretion.

In 20-week-old *car14*^−/−^ littermates, TUDCA-induced bile flow was non significantly reduced in *car14*^−/−^ compared to *car14*^+*/*+^ mice, while it was significantly reduced in 1-year-old *car14*^−/−^ mice compared to the respective littermates (Fig. 2B, C).

### Car14 deficiency induces a mild peripheral bile duct proliferation and fibrosis at advanced age

**Fig. 3:**
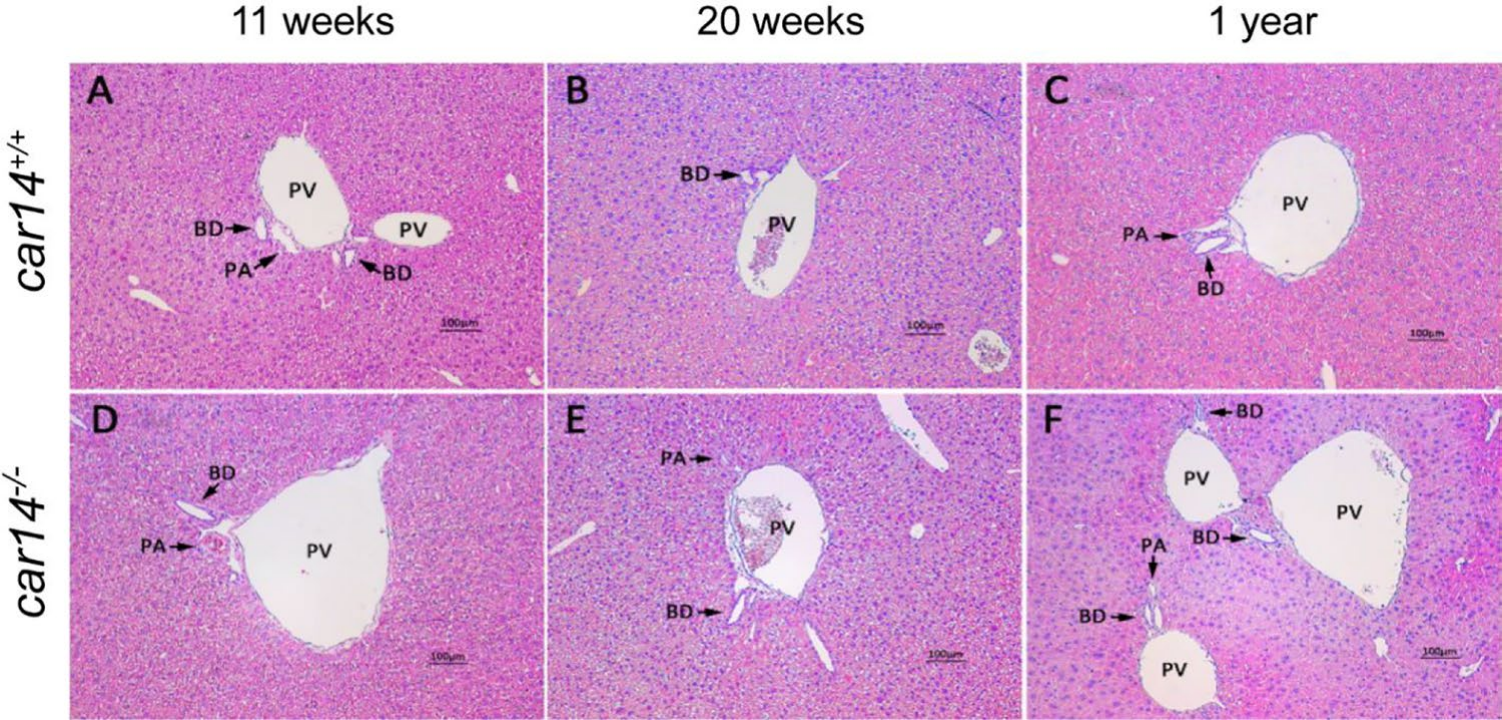
Normal liver histology by hematoxylin and eosin staining in *car14*^*−/−*^ and *car14*^+*/*+^ littermates. (**A**–**F**) H&E staining of liver sections did not reveal conspicuous abnormalities over the observed lifespan of the mice. BD bile duct, PA portal artery, PV portal vein. Representative images of *n* = 4–7 in each group

**Fig. 4:**
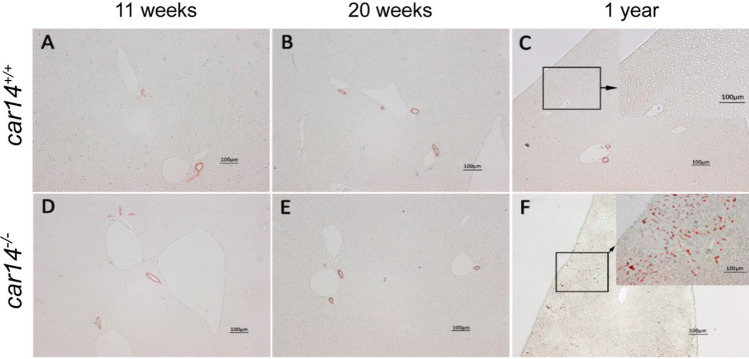
Immunohistochemical staining of cytokeratin 19 did not show obvious differences of the liver sections of *car14*^*−/−*^ and *car14*^+*/*+^ littermates at 11 weeks (**A**, **D**) and 20 weeks (**B**, **E**). (**C**) Compared to the levels in the *car14*^+*/*+^, Cytokeratin 19 staining showed increased staining (**F**) of small ducts in the liver periphery at 52 weeks. Representative images of *n* = 4–7 in each group

**Fig. 5:**
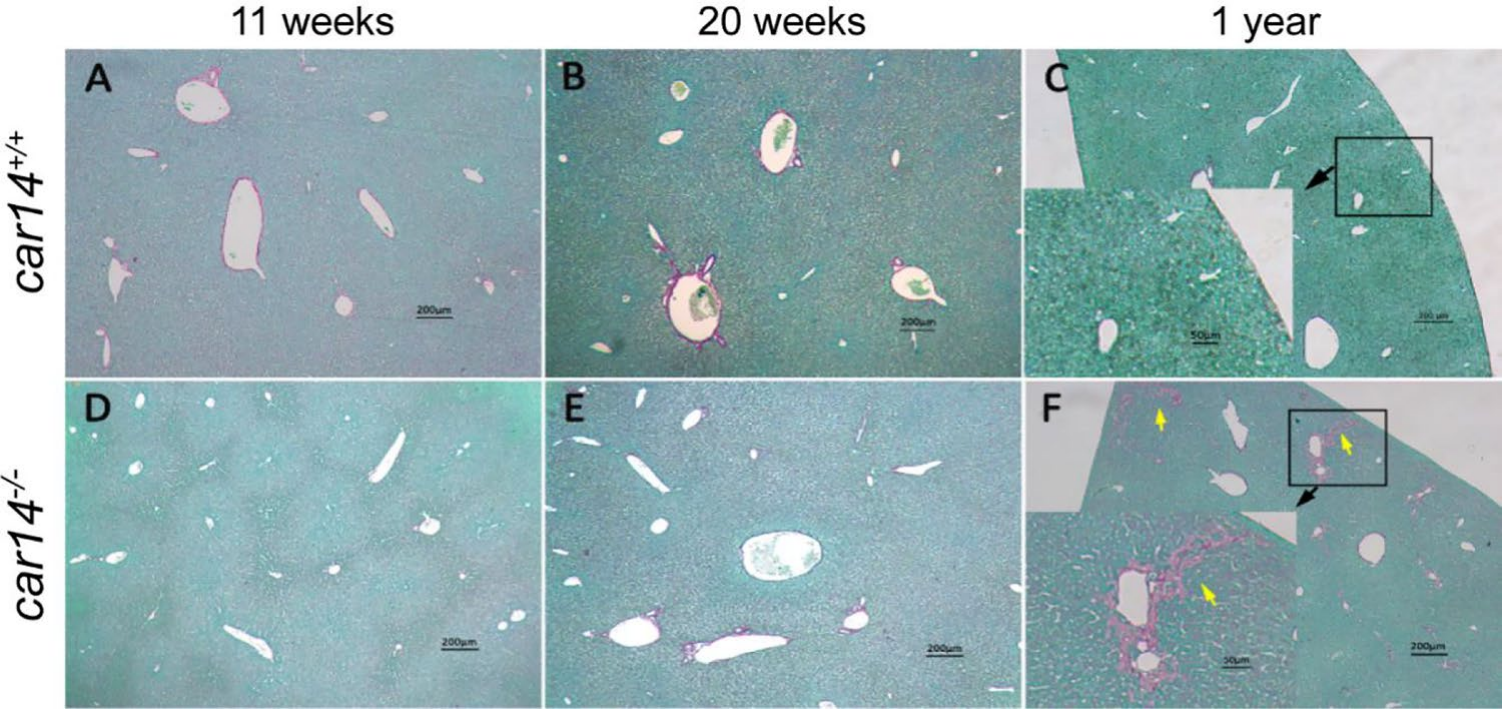
Sirius Red stain for liver fibrosis did not show obvious differences of the liver sections of *car14*^*−/−*^ and *car14*^+*/*+^ littermates at 11 weeks (**A**, **D**) and 20 weeks (**B**, **E**). (**C**) Unlike in the *car14*^+*/*+^, a significant increase of the magenta staining indicating increased collagen content (**F**) was observed in the liver periphery at 52 weeks. Representative images of *n* = 4–7 in each group

The progressive nature of the reduction in HCO_3_^−^ output as well as bile flow in the absence of Car14 suggests structural changes as a potential cause. At young age, histological evaluation revealed no differences between *car14*^−/−^ and *car14*^+*/*+^ mice (Fig. 3A–F). Even at advanced age, no obvious differences in H&E staining in *car14*^−/−^ and *car14*^+*/*+^ mice were seen (Fig. 3A–F). However, staining for cytokeratin 19, a marker for bile ducts, showed an increased staining of the small bile ducts in the liver periphery in 1-year-old mice (Fig. 4A–F). In addition, in the Sirius Red staining for collagen, mild fibrosis was seen in the liver periphery of 1-year-old *car14*^−/−^ compared to *car14*^+*/*+^ mice (Fig. 5A–F). Thus, a reduction of small bile duct frequency and diameter may be a reason for the reduction in bile flow in *car14*^−/−^ compared to *car14*^+*/*+^ mice at advanced age.

**Fig. 6:**
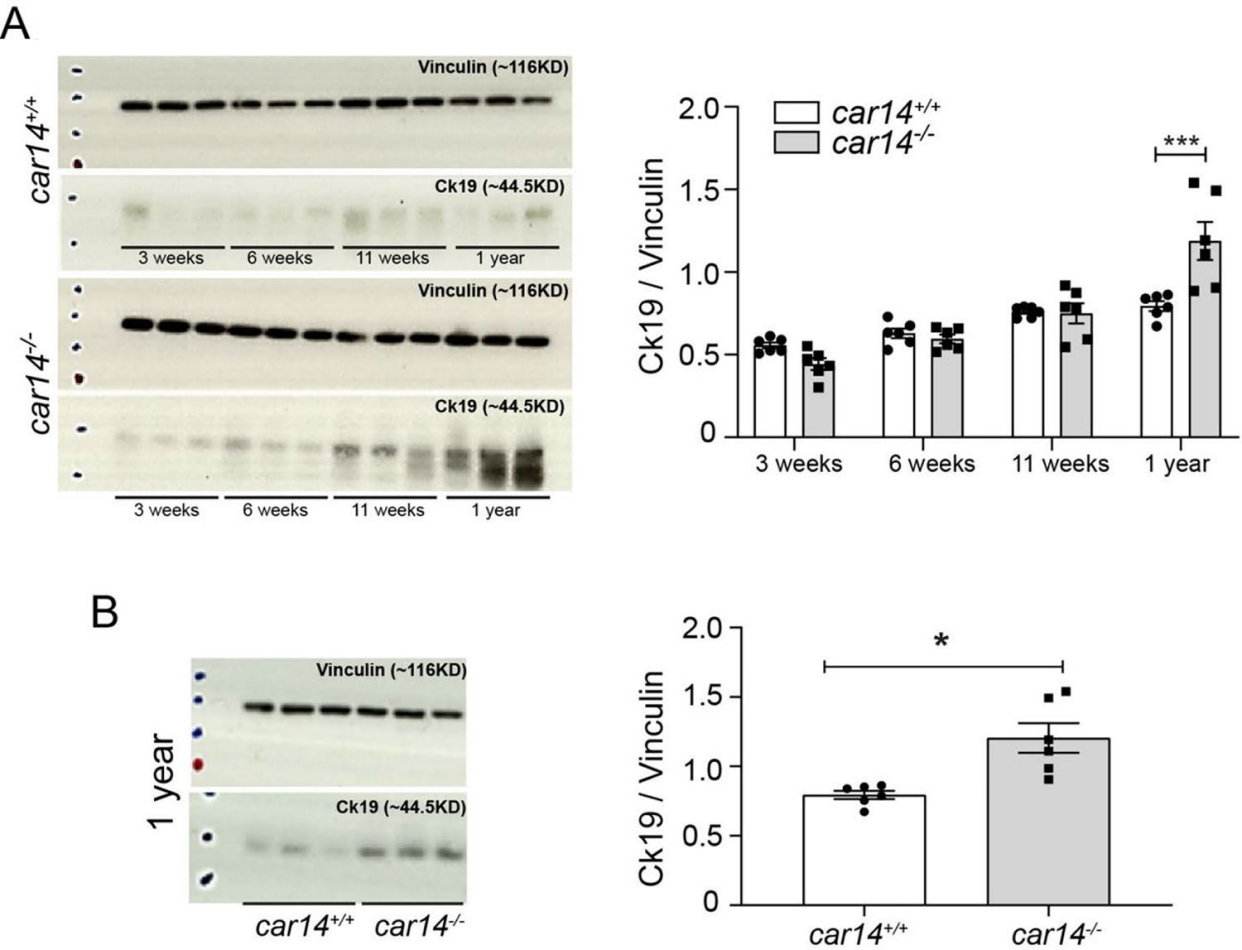
Car14 deficiency induces a mild peripheral bile duct proliferation and fibrosis at advanced age. Western analysis of liver tissue (lobe 1 + 4) from 3, 6, 11, and 52 weeks/age of *car14*^*−/−*^ and *car14*^+*/*+^ littermates. (**A**) Representative image and the summary of a total of 6 individual samples for lobe 1b + 4 at different age, and (**B**) representative and the summary of a total of 6 whole liver homogenates at 1 year of age. In both instances, a significant upregulation of cytokeratin 19 in *car14*^*−/−*^ liver compared to that of *car14*^+*/*+^ littermates is seen at 1 year of age

In order to verify the histological results biochemically, we performed Western analysis of liver tissue (lobe 1 + 4) from 3, 6, 11, and 52 weeks of *car14*^−/−^ and *car14*^+*/*+^ littermates. Figure 6A displays a representative image and the summary of a total of 6 individual samples for lobe 1b + 4 (probably more material from the liver periphery than in Fig. 5B) at different age, and Fig. 6B a representative and the summary of a total of 6 whole liver homogenates at 1 year. In both instances, a significant upregulation of cytokeratin 19 in *car14*^−/−^ liver compared to that of *car14*^+*/*+^ littermates is seen at 1 year of age.

### Car14 deficiency does not result in an increase in pro-inflammatory cytokine production

**Fig. 7:**
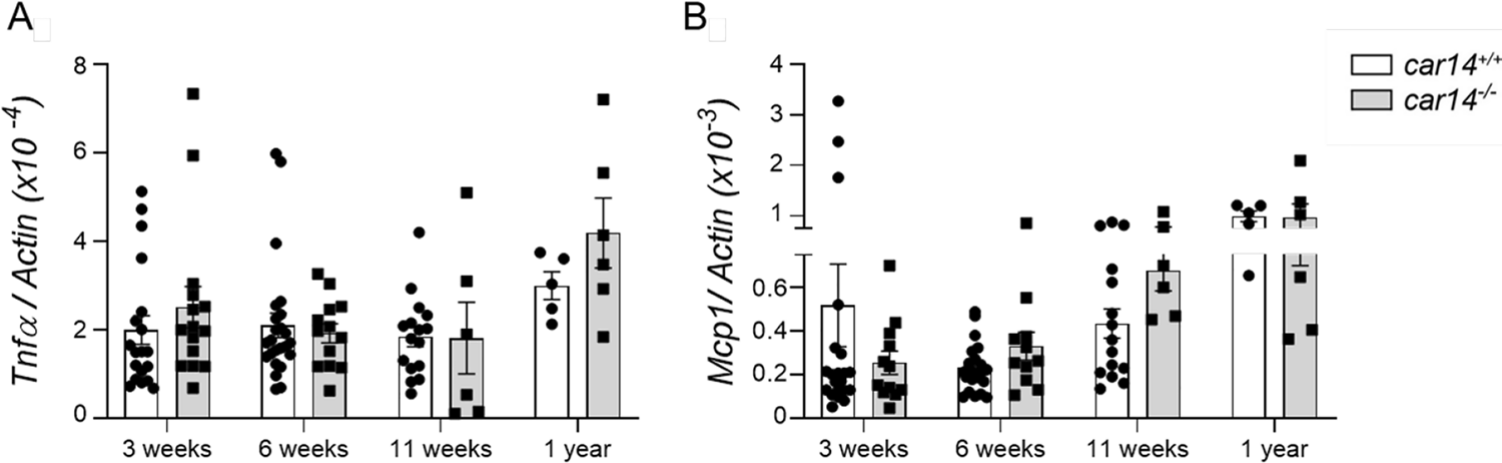
Car14 deficiency does not result in an increase in pro-inflammatory cytokine production. *Tnfα* mRNA (**A**) and *Mcp1* mRNA (monocyte chemotactic protein 1, CCL2) (**B**) expression in *car14*^*−/−*^ and *car14*^+*/*+^ livers from 3 weeks to 1 year of age. No significant differences were observed in any age group

Intraluminal acidity in conjunction with bile acids is considered to damage epithelia. We therefore measured the expression for *Tnf-α* and *Mcp-1* (monocyte chemotactic protein 1, CCL2), which is elevated early in the liver of *mdr2*^−/−^ mice. There was no significant difference in mRNA expression of the two cytokines in *car14*^−/−^ and *car14*^+*/*+^ mice from 3 weeks to 1 year of age (Fig. 7A, B).

## Discussion

Bile acids form mixed micelles with phospholipids and cholesterol in hepatic bile, which greatly reduces their ability to damage membranes. In addition, the formation of mixed micelles enables the hepatocytes to excrete the water-insoluble cholesterol into bile and thus into the intestine [24]. The “critical micellar concentration” of bile acids is pH-dependent [25]. Therefore, the regulation of the biliary pH is of great physiological relevance. Soon after fluorescent pH_i_ indicators had been discovered, the mechanisms of acid/base transport and intracellular pH regulation were studied in isolated and cultured hepatocytes or “hepatocyte couplets,” which still express a distinct canalicular membrane [21, 23, 43].

Biliary alkaline output requires acid and base extrusion mechanisms both the sinusoidal and the canalicular membrane of the hepatocyte. Early work in isolated canalicular membrane vesicles described the presence of a Cl^−^/HCO_3_^−^ exchanger [35], a finding that has been verified in hepatocyte couplets [8]. In addition, hepatocyte basolateral membrane vesicles were isolated to functionally demonstrate the presence of Na^+^/H^+^ exchangers [5] as well as Na^+^/HCO_3_^−^ cotransporters [44] in the hepatocyte basolateral membranes. Interestingly, this hepatocyte apical anion exchanger was characterized as AE2 (SLC4A4), which is basolaterally sorted in other epithelia [4, 33]. However, the presence of anion exchangers from the SLC26 family of multifunctional anion transporters in the canalicular membrane has also been described [9, 27]. In addition, the CFTR anion channel, as well as Ca^2+^-dependent anion channels, is believed to be involved in the regulation of biliary fluid and HCO_3_^−^ output [2]. CFTR has been localized in cholangiocytes but not hepatocytes [13]. Studies in the mouse liver revealed an expression of CFTR, secretin receptors, and AE2 anion exchanger in large hepatobiliary ducts [20]. While secretin-stimulated bile flow is CFTR-dependent, bile acid–stimulated bile flow is not [15].

In pilot experiments, we had measured basal, secretin-stimulated, and TUDCA-stimulated bile flow and biliary HCO_3_^−^ output. TUDCA infusion stimulated a markedly higher rate of bile flow and HCO_3_^−^ output than secretin in the applied doses, suggesting that the origin of the HCO_3_^−^ and fluid that we measure is predominantly hepatocellular. This is in accordance with the view that $\sim$ 90% of hepatic bile in rodents originates from the hepatocytes, and only $\sim$ 10% is of cholangiocellular origin [13].

**Fig. 8:**
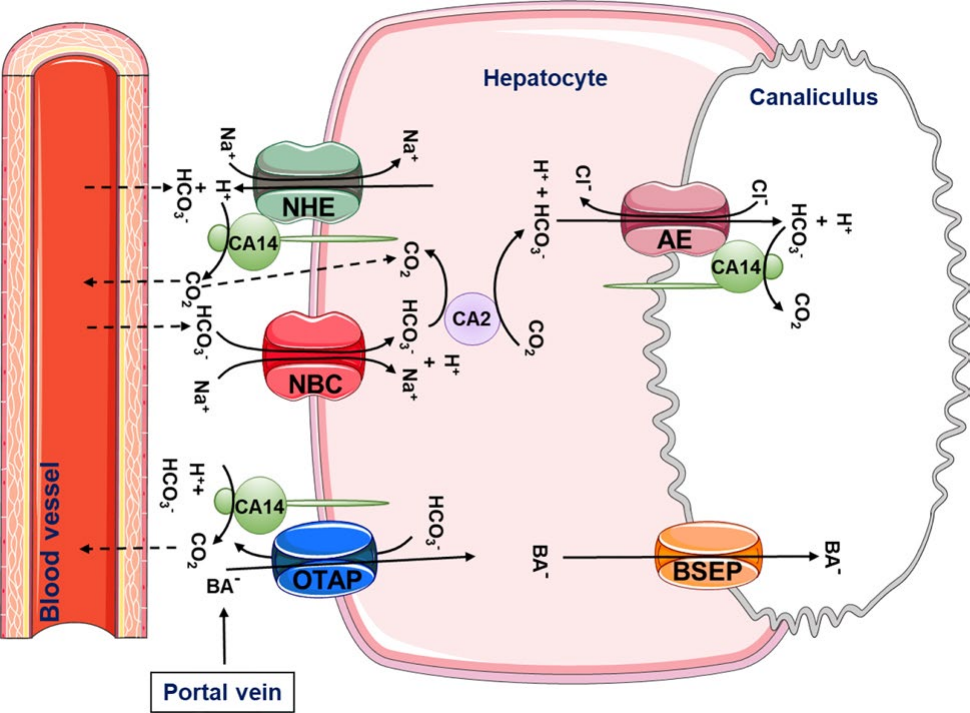
Cartoon depicting the speculative role of Car14 (CAXIV) in the maintenance of biliary alkalinity. At the sinusoidal membrane, Car14 may increase the rate of proton dissipation from the external binding site of the Na^+^/H^+^ exchanger and thus serve in stabilizing an alkaline intracellular pH (pH_i_). Car14 may also increase the rate of TUDCA/HCO_3_^*−*^ exchange (speculative). At the canalicular membrane, Car14 may increase the rate of canalicular AE2-mediated HCO_3_^*−*^ export to the lumen by rapidly removing HCO_3_^*−*^ from the external transport site. Intracellular carbonic anhydrases, most likely Car 2 (CAII), will be tethered to the respective membranes and increase the rate of supply of protons or base to the intracellular binding site of the Na^+^/H^+^ and Cl^*−*^/HCO_3_^*−*^ exchanger, and will increase the rate of HCO_3_^*−*^ removal from the intracellular binding site of Na^+^/HCO_3_^*−*^ cotransporters

Because strong, predominantly canalicular Car14 expression was shown in murine hepatocytes [38], we chose to study the effect of Car14 deletion on basal and taurocholate-induced HCO_3_^−^ output and bile flow rates in young, middle age and aged mice. Biliary HCO_3_^−^ output rates were highest in young mice, and Car-14 deletion resulted in significantly lower TUDCA-induced increase in biliary HCO_3_^−^ output compared to that in *car14*^+*/*+^ littermates in all age groups (Fig. 1). Interestingly, bile flow rates were not affected by Car14 deletion at young age (Fig. 2A), suggesting that the presence of Car14 enhances HCO_3_^−^ output via an anion exchanger rather than an anion channel, most likely expressed in the canalicular membrane. However, Car14 immunostaining was also detected in the sinusiodal membrane, albeit with weaker intensity [40]. A basolateral Car14 with its catalytic site in the extracellular mileau may serve to enhance proton dissipation from the external binding site of Na^+^/H^+^ exchangers and enhance the rate of proton extrusion. This will facilitate the maintenance of an alkaline intracellular pH and thus a higher rate of apical HCO_3_^−^ export [38]. In addition, it is feasible that Car14 operates in conjunction with basolateral OATPs, which absorb TUDCA (and other organic anions) in conjunction with HCO_3_^−^ or glutation (GST) exchange [49]. A rapid removal of HCO_3_^−^ from the external transport site of OATPs may also increase the transport rate through these transporters; however, this is speculative. The same holds true for other hepatic transporters. A schematic diagram is given in Fig. 8.

At higher age, the basal and TUDCA-stimulated HCO_3_^−^ output rates decreased in both *car14*^−/−^ and *car14*^+*/*+^ mice, but also the percentage of TUDCA-induced increase in HCO_3_^−^ output rate decreased significantly in *car14*^−/−^ compared to *car14*^+*/*+^ mice (Fig. 1B, C). In addition, the TUDCA-stimulated bile flow rates also decreased with advanced age (Fig. 2B, C). One potential explanation for this finding was the development of structural changes in the liver and/or biliary system. We therefore investigated the liver histology. We could not detect histological alterations in H&E staining between *car14*^−/−^ and *car14*^+*/*+^ liver (Fig. 3), but immunohistochemical staining for cytokeratin 19, a marker for the cholangiocytes, revealed increased staining at the liver lobe periphery, seen in 1-year-old mice (Fig. 4F). In addition, peripheral mild fibrotic changes were observed in the Sirius Red stain. Western analysis confirmed the increase in cytokeratin-19 expression and thus a mild proliferation of small bile ducts in the liver periphery (Fig. 6). This suggests that the absence of Car14, possibly via a chronically low biliary HCO_3_^−^ output, results in the development of a mild fibrotic phenotype in the liver.

Cholangiocyte proliferation and fibrosis is also a feature of mouse models of sclerosing cholangitis such as the *mdr2*^−/−^ mouse [51]. Consistent with previous publications [16, 28, 41], the changes that we observed in the liver of *mdr2*^−/−^ mice on the same genetic background and with the same breeding conditions were observed much earlier and were much more severe than in the *car14*^−/−^ liver. In a recent review, Trauner et al. list the various causes for “toxic” bile formation, among them an increased bile acid/phospholipid ratio, as is the case in patients with MDR3 mutations or the *mdr2*^−/−^ mouse, or a decreased HCO_3_^−^ concentration and bile hydration, such as in cystic fibrosis patients and in the *cftr*^−*/*−^ mouse, or possibly due to AE2 downregulation in primary biliary cirrchosis [50]. The *car14*^−/−^ mouse model belongs to the latter category, but displays a mild phenotype. Disease-causing mutations in human carbonic anhydrase isoforms are known, but not yet for Car14.

In summary, the *car14*^−*/*−^ mouse model displays a selective decrease in biliary HCO_3_^−^ concentration at young age, prior to the development of a mild sclerosing cholangiopathy. Due to its staining pattern, the membrane-bound Car14 may serve to enhance acid/base transport both in the sinusoidal, but particularly at the canalicular hepatocyte membrane. This may serve to optimize the maintenance of hepatocyte steady-state pH_i_, but also, importantly, may serve to rapidly remove acid moieties in the lumen and thereby alkalinize the biliary pH. The higher acidity of the *car14*^−/−^ bile is able to slowly induce structural changes in the liver and a reduction in bile flow.

## Authorship contributions

Z.Z., J.Q., B.R., and U.S. designed, analyzed, and performed experiments, A.K. analyzed data and constructed the graphic layout, D.R and U.S planned and supervised the mouse breeding, G.G. provided the Car14-deficient mouse strain and made insightful suggestions, and U.S wrote the manuscript.

## Supplementary Information

Below is the link to the electronic supplementary material.

Supplementary file1 (DOCX 22 KB)
