# Supplementary material for: Loss of luminal carbonic anhydrase XIV results in decreased biliary bicarbonate output, liver fibrosis, and cholangiocyte proliferation in mice
Source: Pflugers Arch. 2022 Feb 4;474(5):529–39. doi: 10.1007/s00424-021-02659-3 (PMC8993780; doi:10.1007/s00424-021-02659-3)
Supplement: Supplementary file 1 — Supplementary file1 (DOCX 22 KB) [file 424_2021_2659_MOESM1_ESM.docx]

**Supplementary Table 1: List of Primers**

| **Gene** | **Forward** | **Reverse** | **Company** |
| --- | --- | --- | --- |
| *Actin* | AGAGGGAAATCGTGCGTGAC | CAATAGTGATGACCTGGCCGT | Sigma |
| *Mcp1* | CTGCTGTTCACAGTTGCCG | GCACAGACCTCTCTCTTGAGC | Sigma |

| **Gene** | **Company / Catalog number** |
| --- | --- |
| *Tnfα* | Qiagen , Quantitect # QT00104006 |
